# Preoperative Serum Cortisol Level Is Predictive of Weight Loss After Laparoscopic Sleeve Gastrectomy in Men with Severe Obesity but Not Women

**DOI:** 10.1007/s11695-022-06415-z

**Published:** 2023-01-10

**Authors:** Hironori Bando, Hiroshi Miura, Seiichi Kitahama, Shinsuke Nakajima, Tetsuya Takahashi, Toshihiko Mihara, Teppei Momono, Maki Kimura-Koyanagi, Kazuhiko Sakaguchi, Tomoichiro Mukai, Wataru Ogawa, Yoshikazu Tamori

**Affiliations:** 1 Division of Diabetes and Endocrinology, Department of Internal Medicine, Kobe University Graduate School of Medicine, Kobe, Japan; 2 Department of Metabolic and Bariatric Surgery, Center for Obesity, Diabetes, and Endocrinology, Chibune General Hospital, Osaka, Japan; 3 Department of Diabetes and Endocrinology, Center for Obesity, Diabetes, and Endocrinology, Chibune General Hospital, Osaka, Japan; 4 Department of General Surgery, Chibune General Hospital, Osaka, Japan; 5 Division of Medical Education, Department of Internal Medicine, Kobe University Graduate School of Medicine, Kobe, Japan; 6 Division of General Internal Medicine, Department of Internal Medicine, Kobe University Graduate School of Medicine, Kobe, Japan; 7 Division of Creative Health Promotion, Department of Social/Community Medicine and Health Science, Kobe University Graduate School of Medicine, 7-5-1 Kusunoki-Cho, Chuo-Ku, Kobe, 650-0017 Japan

**Keywords:** Bariatric surgery, Cortisol, Obesity, Total weight loss

## Abstract

**Background:**

Bariatric surgery is an effective treatment for severe obesity and its associated medical problems. Preoperative factors that predict postoperative weight loss remain to be fully characterized, however.

**Methods:**

Anthropometric and laboratory data were collected retrospectively for severely obese patients who underwent laparoscopic sleeve gastrectomy (LSG) between April 2016 and July 2019 at our hospital. Preoperative factors that predicted weight loss at 1 year after LSG were investigated.

**Results:**

A total of 122 subjects (45 men and 77 women) underwent LSG. The mean ± SD age and body mass index at surgery were 44.4 ± 10.4 years and 40.7 ± 6.7 kg/m^2^. The percent total weight loss (%TWL) was 27.0 ± 8.6 among all subjects, 26.4 ± 8.0 among men, and 27.4 ± 8.9 among women, with no significant difference between the sexes. The %TWL showed a significant inverse correlation with serum cortisol level in men and with age and the visceral/subcutaneous fat area ratio in women. Multivariable regression analysis revealed the presence of type 2 diabetes and the serum cortisol concentration to be negatively associated with %TWL among all subjects and men, respectively. Receiver operating characteristic curve analysis identified an optimal cutoff of 10 µg/dL for prediction of a %TWL of ≥ 25 in men by serum cortisol level.

**Conclusions:**

Serum cortisol concentration was identified as a predictor for postoperative weight loss in men. Our results may thus help inform the decision to perform LSG or more effective surgical procedures in men with severe obesity.

**Graphical Abstract:**

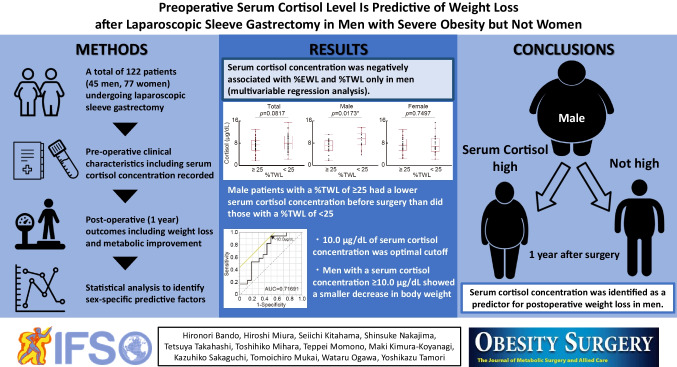

## Introduction

Obesity triggers a wide variety of health problems and thus leads to a decline in the quality and expectancy of life [1]. Whereas treatment of obesity remains a clinical challenge, the mortality of obese individuals decreases with weight loss regardless of the treatment approach, either surgical or nonsurgical [2]. In the case of severe obesity, for which the effect of antiobesity medications is limited, surgery is an effective and reliable treatment option [3].

Bariatric surgery is effective not only for weight loss but also for associated medical problems of obesity such as type 2 diabetes mellitus (T2DM) [4], and it has been rapidly and widely adopted as a treatment for severe obesity—in particular, since the introduction of lower-risk laparoscopic procedures [5]. Approximately 700,000 bariatric surgeries are now performed annually worldwide [6]. Laparoscopic sleeve gastrectomy (LSG) is one of the most commonly performed types of bariatric surgery, given its simplicity of procedure and good outcomes [7].

Several factors have been found to be associated with weight loss after LSG, including body mass index (BMI), age, sex, and comorbid T2DM [8–14]. The pattern of fat accumulation differs between the sexes [15], however, suggesting that the pathophysiology as well as the efficacy of treatment for obesity might also differ. As far as we are aware, no previous study has analyzed sex-specific predictive factors for postoperative weight loss in individuals who undergo bariatric surgery.

The number of bariatric surgeries performed in Japan has been increasing rapidly but is still small compared with that in Western countries [16]. Vulnerability to obesity and its associated medical problems appears to differ among ethnicities [17], and information relating to factors that influence the outcome of bariatric surgery in Japanese individuals is limited. Our aim is therefore identifying sex-specific predictive factors to develop individualized treatment strategies for efficient weight loss for severe obesity in Japanese. We have now performed a retrospective study to examine preoperative anthropometric and laboratory data for 122 Japanese individuals with severe obesity who underwent LSG in an attempt to identify sex-specific predictors of postoperative weight loss.

## Materials and Methods

### Patients

This retrospective observational study was approved by the institutional review board of our hospital (approval no. 20211111A) and conformed to the provisions of the Declaration of Helsinki (as revised in 2013). The study subjects were all patients who underwent LSG at our hospital by a bariatric fellowship-trained surgeon in the setting of a comprehensive multidisciplinary program between April 2016 and July 2019. All participants had a BMI of ≥ 35 kg/m^2^ with obesity-related medical problems and failed to achieve a substantial weight reduction despite more than 6 months of medical therapy. They provided written informed consent for the collection and use of their data for research purposes only. Patients were excluded if they had Cushing’s syndrome or other hormonal abnormalities, or if they had received steroid treatment by multiple endocrinologists. We defined the presence of diabetes mellitus as the taking of hypoglycemic agents or as a fasting plasma glucose concentration of ≥ 126 mg/dL and a hemoglobin A_1c_ (HbA_1c_) level of ≥ 6.5%, according to the diagnostic criteria of the Japan Diabetes Society [18]. T2DM was diagnosed by confirming the absence of antibodies to glutamic acid decarboxylase as well as by excluding diabetes due to other specific mechanisms or diseases in subjects with diabetes mellitus.

### Study Design and Measurements

Anthropometric and laboratory data were obtained immediately before and 1 year after the surgery. The serum cortisol concentration was measured in the morning before breakfast and at rest during hospitalization for surgery. The amount of skeletal muscle and fat mass were measured by the bioelectrical impedance method with an In-Body S20 body composition analyzer (Biospace, Seoul, Korea). The subcutaneous fat area (SFA) and visceral fat area (VFA) were calculated from abdominal computed tomography images obtained at the navel level. The visceral/subcutaneous fat area ratio (VSR) was obtained as 100 × VFA/SFA. The percent total weight loss (%TWL) was calculated as 100 × (operative weight − follow-up weight) / operative weight. The percent excess weight loss (%EWL) was calculated as 100 × (operative weight − follow-up weight) / (operative weight − ideal body weight). The skeletal muscle index (SMI) was calculated by dividing skeletal muscle weight (kg) by body weight (kg). The primary end point of the study was the identification of significant preoperative predictors of %TWL.

### Surgical Procedures

The abdomen was entered under direct vision with a forward-viewing trocar. A 5-mm, three 12-mm, and one 15-mm ports were placed. A Nathanson liver retractor was used to elevate the liver. We mobilized the periesophageal fat pad to visually position the stapler to leave approximately 1 cm of gastric tissue lateral to the angle of His. The pylorus was identified, and an area approximately 4 cm from the pylorus was chosen to begin ligating and transecting the greater curvature vessels with a vessel-sealer device. The greater curvature of the stomach was mobilized to the angle of His, with particular attention paid to mobilizing the entire fundus to the mid-portion of the left crura of the diaphragm. A 34-French bougie was passed by an anesthesiologist and positioned in the distal antrum. Resection of the antrum was started tangentially from the right lateral port using a linear stapler, positioning the tip of the stapler to give a distance of 1 cm from the bougie at the area of the incisura angularis. Resection of the body and fundus of the stomach was achieved via the 12-mm left mid-clavicular port site to the angle of His. It was our practice to oversew the staple line with 2–0 nonabsorbable suture. The 12- and 15-mm port sites were closed with absorbable sutures.

### Statistical Analysis

All statistical analysis was performed with the use of JMP Statistical Database Software version 12.2.0 (SAS Institute, Cary, NC, USA). Analysis of variance (ANOVA), correlation analysis, multivariable regression analysis, and receiver operating characteristic (ROC) curve analysis were performed as appropriate. Multivariable regression analysis was performed to identify potential independent predictors of postsurgery weight loss. Age, BMI, SMI, VSR, T2DM, and serum cortisol level were included as covariates. These factors were selected as explanatory covariates because aging is associated with changes in body composition and physical function [19], BMI is associated with obesity-related outcomes [20], the amount of skeletal muscle is associated with energy expenditure [21], T2DM is associated with difficulty in losing body weight [22], insufficient weight loss in bariatric surgery was associated with a history of hypertension [23], and cortisol promotes body weight gain and obesity [24]. VSR is known to be different between the sexes [15] and has a strong association with cardiometabolic risks [25], but its effect on weight loss is unclear. All reported *P* values are two-tailed, and those of < 0.05 were considered statistically significant.

## Results

### Patient Characteristics and Effectiveness of LSG

A total of 122 severely obese individuals (45 men and 77 women) underwent LSG. The characteristics of these study patients are shown in Table 1. The mean ± SD age and BMI at the time of surgery were 44.4 ± 10.4 years and 40.7 ± 6.7 kg/m^2^, and the prevalence of T2DM was 47.5%. BMI had decreased from 40.7 to 28.0 kg/m^2^, and the effectiveness of surgery as evaluated by %TWL was 27.0 ± 8.6 at 1 year after surgery. Whereas skeletal muscle weight decreased after LSG, SMI increased, suggesting that surgery reduced fat composition predominantly. Blood pressure was decreased, and parameters of glucose metabolism were improved after surgery. With regard to lipid metabolism, triglyceride and high-density lipoprotein cholesterol levels were decreased and increased after LSG, respectively. Serum adiponectin and leptin concentrations were increased and decreased, respectively. Analysis according to sex revealed that BMI was decreased in both men and women, and %TWL did not differ between the sexes (26.4 ± 8.0 in men and 27.4 ± 8.9 in women; *p* = 0.5609, Student’s *t*-test). Low-density lipoprotein cholesterol was decreased after surgery only in men. Preoperative serum cortisol concentrations were < 16 μg/dL in all subjects and did not differ between the sexes (*p* = 0.3690, Student’s *t*-test).

**Table 1:** Clinical characteristics of the patients before and after laparoscopic sleeve gastrectomy

	Total (*n* = 122)							Male (*n* = 45)							Female (*n* = 77)						
	Preoperative			Postoperative			*p*-value (pre vs post)	Preoperative			Postoperative			*p*-value (pre vs post)	Preoperative			Postoperative			*p*-value (pre vs post)
Age (years)	44.4 ± 10.4							43.6 ± 9.9							44.7 ± 10.7						
BMI (kg/m^2^)	40.7	±	6.7	28.0	±	5.4	< 0.0001*	42.3	±	8.1^†^	29.7	±	6.0^§^	< 0.0001*	39.7	±	5.6^†^	27.0	±	4.7^§^	< 0.0001*
Body weight (kg)	104	±	20.2	75.7	±	17.4	< 0.0001*	121	±	19.6^†^	89.5	±	17.2^§^	< 0.0001*	93.4	±	12.4^†^	68.1	±	12.2^§^	< 0.0001*
Skeletal muscle weight (kg)	31.5	±	7	28.0	±	6.3	< 0.0001*	39.3	±	4.5^†^	34.9	±	4.7^§^	< 0.0001*	27.2	±	3.3^†^	24.3	±	3.1^§^	< 0.0001*
SMI (%)	30.3	±	3.5	37.5	±	5.3	< 0.0001*	32.6	±	3.8^†^	39.7	±	6.0^§^	< 0.0001*	29.0	±	2.6^†^	36.3	±	4.5^§^	< 0.0001*
SBP (mmHg)	134	±	17.2	121	±	16.6	< 0.0001*	137	±	18.6	124	±	18.5	0.0122*	131.5	±	16	119	±	15.1	0.0003*
DBP (mmHg)	83.1	±	12.6	75.4	±	11.2	0.0004*	83.2	±	14.2	76.7	±	12.0	0.0080*	83.1	±	11.7	74.6	±	10.7	0.0239*
Prevalence of hypertension (%)	72.4							79.1							68.5						
Total fat area (cm^2^)	688	±	185	341	±	161	< 0.0001*	752	±	196.6^†^	369	±	201	< 0.0001*	651.2	±	168.0^†^	325	±	132	< 0.0001*
VSR (%)	50.1	±	25.9	30.6	±	20.7	< 0.0001*	59.6	±	30.0^†^	34.0	±	27.2	< 0.0001*	44.6	±	21.6^†^	28.7	±	15.7	< 0.0001*
Serum total protein (g/dL)	6.96	±	0.4	6.96	±	0.52	0.0692	6.96	±	0.41	7.02	±	0.54	0.8659	7.0	±	0.40	6.92	±	0.50	0.0298*
Prevalence of T2DM (%)	47.5							57.8							41.6						
HbA1c (%)	6.13	±	0.87	5.59	±	0.55	< 0.0001*	6.22	±	1.05	5.48	±	0.59	< 0.0001*	6.1	±	0.74	5.65	±	0.52	< 0.0001*
FPG (mg/dL)	106	±	18.7	95.5	±	16.6	0.0002*	110	±	22.3	100	±	14.2^§^	0.0639	103.5	±	16.0	93.0	±	17.4^§^	0.0012*
Serum TG (mg/dL)	143	±	72.9	90.1	±	46.2	< 0.0001*	157	±	72.2	98.5	±	50.6	< 0.0001*	138.3	±	72.9	85.4	±	43.2	< 0.0001*
Serum CPR (ng/mL)	3.09	±	1.47	1.84	±	0.73	< 0.0001*	3.48	±	1.80^†^	2.15	±	0.86^§^	< 0.0001*	2.9	±	1.19^†^	1.68	±	0.59^§^	< 0.0001*
Serum HDL-Chol (mg/dL)	47.0	±	10.4	72.1	±	19.3	< 0.0001*	44.0	±	10.5^†^	65.7	±	21.7^§^	< 0.0001*	48.8	±	10.0^†^	75.7	±	17.1^§^	< 0.0001*
Serum LDL-Chol (mg/dL)	117	±	43	114	±	33.8	0.3007	112	±	32.3	99.3	±	26.5^§^	0.0173*	120.1	±	34.9	123	±	34.7^§^	0.7478
LDL/HDL-Chol ratio	2.63	±	1.02	1.72	±	0.70	< 0.0001*	2.74	±	1.12	1.73	±	0.8	< 0.0001*	2.6	±	0.96	1.71	±	0.65	< 0.0001*
Serum adiponectin concentration (µg/dL)	6.74	±	3.7	15.2	±	10.4	< 0.0001*	5.95	±	3.21	13.3	±	8.3	< 0.0001*	7.2	±	3.9	16.2	±	11.2	< 0.0001*
Serum leptin concentration (ng/dL)	51.0	±	32.3	17.2	±	12.3	< 0.0001*	42.8	±	26.8^†^	12.3	±	9.9^§^	< 0.0001*	55.7	±	34.4^†^	19.8	±	12.7^§^	< 0.0001*
Serum cortisol concentration (μg/dL)	7.9	±	3.1					8.2	±	3.3					7.7	±	3.0				
%TWL				27.0	±	8.6					26.4	±	8.0					27.4	±	8.9	

Data are means ± SD. *BMI*, body mass index; *SMI*, skeletal muscle index; *SBP*, systolic blood pressure; *DBP*, diastolic blood pressure; *VSR*, visceral/subcutaneous fat area ratio; *T2DM*, type 2 diabetes mellitus; *HbA1c*, glycosylated hemoglobin; *FPG*, fasting plasma glucose; *TG*, triacylglycerol; *CPR*, C-peptide reactivity; *HDL-chol*, high-density lipoprotein cholesterol; *LDL-chol*, low-density lipoprotein cholesterol; *%TWL*, percent total weight loss. *p < 0.05 (preoperative vs postoperative). †*p* < 0.05 (preoperative male vs preoperative female). §*p* < 0.05 (postoperative male vs postoperative female)

### Factors that Correlate with %TWL

The linear correlation between preoperative anthropometric or laboratory parameters and %TWL at 1 year after surgery is shown in Table 2. Among all patients, age (*r* =  − 0.2438, *p* = 0.0122) and VSR (*r* =  − 0.1938, *p* = 0.0487) showed a significant negative correlation with %TWL. Serum cortisol level was significantly and negatively correlated with %TWL in men (*r* =  − 0.4629, *p* = 0.0067), whereas age (*r* =  − 0.3035, *p* = 0.0119) and VSR (*r* =  − 0.2814, *p* = 0.0201) were weakly (but significantly) and negatively correlated with %TWL in women.

**Table 2:** Correlation between preoperative clinical parameters and %TWL at 1 year after laparoscopic sleeve gastrectomy

	Total			Male			Female	
	*r*	*p*-value		*r*	*p*-value		*r*	*p*-value
Age	− 0.2438	0.0122*		− 0.1104	0.5154		− 0.3035	0.0119*
BMI	− 0.0242	0.8067		− 0.0001	0.9536		− 0.0150	0.9035
SMI	− 0.0545	0.5867		0.1012	0.5628		− 0.1604	0.1947
SBP	− 0.0723	0.4772		-0.1195	0.5010		− 0.0336	0.7904
DBP	0.0658	0.5190		− 0.0420	0.8137		0.1220	0.3329
VSR	− 0.1938	0.0487*		− 0.0331	0.8480		− 0.2814	0.0201*
FPG	− 0.0723	0.4638		− 0.0167	0.9207		− 0.1010	0.4123
HbA1c	− 0.1391	0.1589		− 0.1423	0.4008		− 0.1358	0.2374
Adiponectin	0.1839	0.0670		0.0987	0.5726		0.2037	0.1036
Leptin	− 0.0211	0.8356		− 0.1522	0.3903		0.0013	0.9917
CPR	0.0036	0.9708		− 0.0481	0.7773		0.0686	0.5784
TG	− 0.1369	0.1648		− 0.1542	0.3623		− 0.1164	0.3445
HDL-chol	0.1215	0.2170		0.2836	0.0890		0.0262	0.8318
LDL-chol	− 0.0302	0.7597		0.0715	0.6741		− 0.0831	0.5003
Cortisol	− 0.1937	0.0706		− 0.4629	0.0067*		− 0.0491	0.7217

*BMI*, body mass index; *SMI*, skeletal muscle index; *SBP*, systolic blood pressure; *DBP*, diastolic blood pressure; *VSR*, visceral/subcutaneous fat area ratio; *FPG*, fasting plasma glucose; *HbA1c*, glycosylated hemoglobin; *CPR*, C-peptide reactivity; *TG*, triacylglycerol; *HDL-chol*, high-density lipoprotein cholesterol; *LDL-chol*, low-density lipoprotein cholesterol; *%TWL*, percent total weight loss. *p-value < 0.05

### Preoperative Factors Predicting Efficient Weight Loss at 1 Year After LSG

To identify independent predictors of weight loss outcome, we performed multivariable regression analysis. We selected seven preoperative factors—age, BMI, SMI, VSR, T2DM, hypertension, and serum cortisol level—as explanatory variables (Table 3). This analysis revealed that T2DM was negatively associated with %TWL among all patients (*p* = 0.0267), whereas the serum cortisol level was negatively associated with %TWL in men (*p* = 0.0215), and there were no independent predictors of %TWL for women.

**Table 3:** Multivariable regression analysis of %TWL and various preoperative clinical parameters

Factors	Total				Male				Female		
	*t*-value	*p*-value	VIF		*t*-value	*p*-value	VIF		*t*-value	*p*-value	VIF
Age	− 1.43	0.1560	1.3045		− 0.29	0.7768	1.4800		− 1.18	0.2429	1.3589
BMI	− 0.46	0.6438	1.3620		− 0.25	0.8015	3.4824		− 0.88	0.3849	1.5464
SMI	− 0.44	0.6633	1.5568		0.12	0.9027	3.5720		− 0.94	0.3537	1.8590
VSR	− 0.40	0.6907	1.4078		− 0.63	0.5382	1.5316		− 0.61	0.5421	1.3589
T2DM	− 2.26	0.0267*	1.3621		− 1.92	0.2853	1.2148		− 0.98	0.3344	1.8160
Hypertension	− 0.07	0.9458	1.0993		− 0.38	0.7114	1.2641		0.02	0.9877	1.2662
Cortisol	− 1.00	0.3187	1.1969		− 2.49	0.0215*	1.3802		− 0.15	0.8840	1.3589

*%TWL*, percent total weight loss; *BMI*, body mass index; *SMI*, skeletal muscle index; *VSR*, visceral/subcutaneous fat area ratio; *T2DM*, type 2 diabetes mellitus; and *VIF*, variance inflation factor. *p-value < 0.05

To confirm the potential of serum cortisol level as a predictor of postoperative body weight in men, we also performed multivariable regression analysis using another weight loss marker, %EWL as well. We found that only serum cortisol level was negatively associated with %EWL in men (*p* = 0.0308), whereas BMI was negatively associated with %EWL in total subjects (*p* =  < 0.0001) and in women (*p* = 0.0009).

**Fig. 1:**
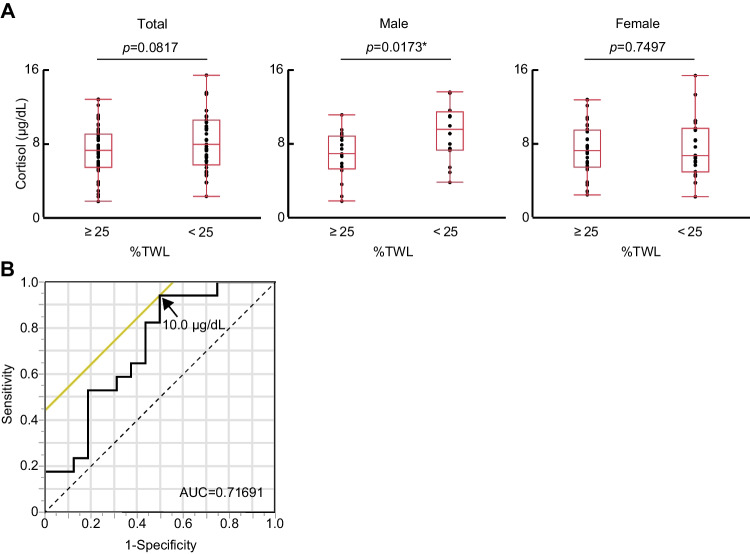
Serum cortisol level before surgery predicts the efficiency of laparoscopic sleeve gastrectomy (LSG) at 1 year after surgery in men. **A** Serum cortisol concentration according to a percent total weight loss (%TWL) cutoff of 25 in all study subjects as well as in male and female subjects separately. The box-and-whisker plots represent the minimum and maximum values (whiskers), the second and third quartiles (box), and the median (midline). *P < 0.05 (Student’s *t*-test). **B** Receiver operating characteristic (ROC) curve analysis of serum cortisol level for prediction of a good response (%TWL of ≥ 25) to LSG in men. The area under the curve (AUC) was 0.717, and the optimal cutoff of 10 µg/dL is indicated

Recent studies have suggested that a good response to bariatric surgery should be defined as a %TWL of ≥ 25 [26, 27]. We, therefore, divided the study patients into two groups with a %TWL of ≥ 25 or < 25. Male patients with a %TWL of ≥ 25 had a lower serum cortisol concentration before surgery than did those with a %TWL of < 25, whereas there was no such difference between the two groups among all patients or women (Fig. 1A). We performed ROC curve analysis to determine the cutoff value of the serum cortisol level for prediction of a good efficacy for LSG (%TWL of ≥ 25) in men. We found that the optimal cutoff was 10.0 µg/dL, which provided a sensitivity of 94.2% and a specificity of 50.0% (Fig. 1B).

### Relation Between Preoperative Serum Cortisol Level and the Metabolic Improvement

Preoperative serum cortisol level was negatively correlated with the decrease of HbA1c (*r* =  − 0.36, *p* = 0.037) and LDL-C (*r* =  − 0.50, *p* = 0.0036) as well as the increase of HDL-C (*r* = 0.48, *p* = 0.0055) at 1 year after surgery only in men. In addition, men with a serum cortisol concentration ≥ 10.0 µg/dL showed smaller improvement in HbA1c level (− 0.17%) after LSG compared to those with cortisol levels < 10.0 µg/dL (− 0.88%) (*p* = 0.038). These data indicated that preoperative cortisol levels were also related to weight loss–induced metabolic improvement after LSG in men.

## Discussion

We have here identified the serum cortisol concentration as a predictive factor for efficient weight loss in Japanese men who underwent LSG. As far as we are aware, our study is the first to analyze sex-specific predictive factors for the outcome of bariatric surgery and to identify serum cortisol level as such a factor.

Cortisol is an obesogenic hormone that stimulates food intake [24]. The reason why the serum level of cortisol was correlated with weight loss only in men is unclear. Estrogen promotes the production of cortisol-binding globulin [28] and thereby alters the ratio of free to total cortisol levels. Given that estrogen levels decline with age after menopause, the total concentration of cortisol measured in the present study might not well reflect the free cortisol level in postmenopausal female subjects. About 35% of the female study subjects were > 50 years of age. A previous study of a Japanese obese cohort consisting of 37 men and 46 women found that the concentration of cortisol in saliva, which reflects well the free cortisol level in serum, was negatively correlated with the extent of weight reduction after nonsurgical treatment [29].

Cortisol concentrations can be measured from hair, saliva, serum, and urine. A recent meta-analysis showed that hair cortisol concentration was associated with adiposity-related outcomes [30]. Measuring hair cortisol is a noninvasive method and stably reflects the long-term effect of cortisol compared with serum cortisol levels which show circadian variations. Hair cortisol concentration may therefore become a more potent predictor for weight loss in the future.

The circulating level of testosterone has been found to be high in men with a low BMI [31], and a reduced level of this hormone is associated with the development and progression of obesity in men [32, 33]. Glucocorticoids inhibit testosterone production by directly influencing Leydig cell function [34, 35]. The serum cortisol concentration may thus affect the outcome of bariatric surgery in men through its effect on testosterone production, given that reduced testosterone enhanced the activity of lipoprotein lipase, resulting in a rise in triacylglycerol uptake to adipose tissues and subsequent obesity [36]. It will thus be of interest to determine whether the circulating testosterone level serves as a predictive factor for the outcome of bariatric surgery in men.

Consistent with previous studies [8, 9, 13, 14], we found that T2DM negatively influenced the extent of weight loss after surgery in the present study. Although the underlying mechanism of this association remains unclear, insufficient adherence to diet therapy might be responsible, as suggested by a previous study [22]. Moreover, certain antidiabetes medications, such as insulin, have been shown to increase weight gain in patients whose diabetes did not improve even after LSG [37]. Whereas previous studies have found that preoperative BMI and age were correlated with weight loss after bariatric surgery [8–13], we did not observe such relations in our study. This apparent discrepancy might be attributable to differences in the number, ethnicity, or male/female ratio of study subjects, in surgical procedure (sleeve gastrectomy, gastric bypass, or gastric banding), in postoperative medical support, or in explanatory variables selected for multivariable regression analysis.

These are several limitations of our study. The study was retrospective in nature, was restricted to a single specialized center, and had a relatively small sample size. In addition, we analyzed only the total cortisol level, not the free cortisol concentration, the latter of which directly reflects the action of the hormone. Furthermore, we did not have data concerning the dexamethasone suppression test, thus could not thoroughly exclude the possibility of autonomous cortisol secretion. We did not evaluate certain clinical parameters that might affect postoperative weight loss, such as mental state, alcohol consumption, diet adherence, and physical activity. In addition, the AUC of the ROC curve analysis was not very robust (0.72). Longer follow-up might increase the robustness of this study. Finally, we cannot exclude possible effects of concurrent medical treatments that reduce body weight, such as diabetes therapy with glucagon-like peptide-1 (GLP-1) receptor agonists and sodium-glucose cotransporter 2 (SGLT2) inhibitors.

## Conclusion

Our data show that the serum cortisol level is an independent predictor of weight loss after LSG in severely obese Japanese men. Such a predictor may be helpful for the choice of surgical procedure, such as LSG or other surgical methods which can be expected to achieve greater weight loss than LSG [38, 39]. Further studies are warranted to clarify the mechanism by which serum cortisol limits the effectiveness of LSG as well as validate the usefulness of cortisol level as a marker for effective weight loss in clinical settings in male patients with severe obesity.

## Data Availability

The data that support the findings of this study are available from the corresponding author upon reasonable request.
